# Hallmarks of peripheral nerve function in bone regeneration

**DOI:** 10.1038/s41413-022-00240-x

**Published:** 2023-01-05

**Authors:** Ranyang Tao, Bobin Mi, Yiqiang Hu, Sien Lin, Yuan Xiong, Xuan Lu, Adriana C. Panayi, Gang Li, Guohui Liu

**Affiliations:** 1 Department of Orthopaedics, Union Hospital, Tongji Medical College, Huazhong University of Science and Technology, Wuhan, 430022 P.R. China; 2 Hubei Province Key Laboratory of Oral and Maxillofacial Development and Regeneration, Wuhan, 430022 P. R. China; 3 Department of Orthopaedics & Traumatology, Stem Cells and Regenerative Medicine Laboratory, Li Ka Shing Institute of Health Sciences, The Chinese University of Hong Kong, Prince of Wales Hospital, Shatin, Hong Kong, SAR 999077 P. R. China; 4 Division of Plastic Surgery, Brigham and Women’s Hospital, Harvard Medical School, Boston, 02215 MA USA

**Keywords:** Bone, Diseases

## Abstract

Skeletal tissue is highly innervated. Although different types of nerves have been recently identified in the bone, the crosstalk between bone and nerves remains unclear. In this review, we outline the role of the peripheral nervous system (PNS) in bone regeneration following injury. We first introduce the conserved role of nerves in tissue regeneration in species ranging from amphibians to mammals. We then present the distribution of the PNS in the skeletal system under physiological conditions, fractures, or regeneration. Furthermore, we summarize the ways in which the PNS communicates with bone-lineage cells, the vasculature, and immune cells in the bone microenvironment. Based on this comprehensive and timely review, we conclude that the PNS regulates bone regeneration through neuropeptides or neurotransmitters and cells in the peripheral nerves. An in-depth understanding of the roles of peripheral nerves in bone regeneration will inform the development of new strategies based on bone-nerve crosstalk in promoting bone repair and regeneration.

## Introduction

Different species have developed unique biological functions that allow them to survive in specific environments through evolution. The ability of most organisms to regenerate or repair tissue after injury or loss has also been significantly impacted by natural selection. In contrast to anthropocentric thinking, animals that possess the capacity for regeneration did not seem to obtain enough evolutionary advantages to make this trait highly conserved. The ability to regenerate is widely but not uniformly distributed among different species.^1^ Some organisms, such as teleost fishes, can regenerate all severed appendages and even vital organs, such as the heart, while many other species, including humans, cannot.^2–4^

In addition to stem cells, which are well-known players, many animal studies of tissue regeneration have suggested the important roles of peripheral nerves in the regeneration of various tissues. Peripheral nerves can be functionally divided into three categories: the autonomic nervous system (ANS), the somatic nervous system, and the enteric nervous system.^5^ Peripheral nerves classically function as links between central and peripheral organs through ligands secreted by terminal axons, establishing a pathway for central-peripheral communication and allowing the central nervous system (CNS) to perceive the external environment. Numerous studies have demonstrated that the ANS and somatic nervous system may be linked to the regeneration process, while the enteric nervous system has been recently shown to play an important role in the regulation of intestinal homeostasis and mucosal regeneration.^6,7^ Nerve fibers in each fascicle are protected by a connective tissue called endoneurium, which contains many cells, such as fibroblasts, macrophages, and vasculature-associated cells.^8^ There is increasing interest in the contributions of resident cells in nerves, Schwann cells (SCs), and endoneurial mesenchymal cells, as well as the nonclassical functions of peripheral nerves, such as the regulation of homeostasis,^9–12^ effects on development,^13,14^ and roles in tissue regeneration.^15–18^

The role of peripheral nerves in regeneration was first discovered in salamander limb regeneration,^19^ which is one of the most common models in regenerative medicine.^20^ Salamander limb regeneration is often considered to reproduce part of the developmental process. The process of regeneration is initiated by wound closure through the wound epithelium (WE). Under the newly formed WE, stump cells dedifferentiate and proliferate to form a blastema, which is a collection of various types of stem cells or progenitor cells. Later, under precise control, the distal blastema forms the apical ectodermal cap and gradually differentiates into a new limb.^20,21^ As the close connection between limb regeneration and the peripheral nervous system (PNS) in the salamander was gradually explored,^21,22^ revealing that reinnervation is indispensable for the restoration of lost or damaged tissues, more attention has been given to the role of the PNS in regeneration, particularly in humans.

Although many mammals, including humans, lack the ability to reconstruct a severed limb, their bone tissue can recover from trauma without scar formation. Bone fracture healing proceeds through four phases: hematoma, soft callus, hard callus, and hard callus remodeling.^23^ Bone healing starts with inflammation resulting from high-energy trauma, and immediate activation of the coagulation cascade leads to hematoma formation at the injured site. With the gradual resolution of inflammation, intramembranous ossification occurs at the periosteum where there is a good blood supply close to the fracture site, whereas areas under hypoxic conditions undergo endochondral ossification. The differentiation of mesenchymal stromal cells (MSCs) from marrow, muscle, and especially the periosteum into bone-lineage cells drives the formation of callus. Finally, the dynamic balance of osteoblast and osteoclast activity remodels newly formed woven bone into lamellar bone. Bones are richly innervated by peripheral nerves, and parallel findings on the role of the PNS in limb regeneration in distant spices and bone regeneration point to the common role of nerves in regeneration.^24,25^ Despite the fact that the role of the PNS in many other processes associated with bone metabolism has not been fully characterized, the inevitable question now applies to bone regeneration: how does communication occur between the PNS and other tissues in the bone regenerative microenvironment?

In this review, we focus on the conserved role of nerves in regeneration during evolution and summarize the innervation of bone under normal physiological conditions and during bone regeneration following trauma. We emphasize the presence of neuro-skeletal, neurovascular and neuroimmune interactions at different stages of bone regeneration and discuss the possible nerve-associated cellular and molecular mechanisms involved in osteogenesis and other processes that are essential for bone formation. We address the limitations and challenges in current studies with the hope of inspiring further research.

## Function of peripheral nerves in tissue regeneration

Because the regeneration of amputated limbs in salamanders, from the blastema to the entire appendage, reflects the ideal outcome of regenerative medicine, efforts have been made to investigate whether the nerve-dependent mechanism is widespread in nature and to identify the animal model that is most similar to humans. In a salamander study, regeneration of the upper limb was inhibited by denervation at the brachial plexus level.^26^ Diverting nerves toward the damaged site could promote limb regeneration^27^ and even the growth of supernumerary limbs.^28^ It is noteworthy that the extent of denervation positively correlated with the impairment of limb regeneration,^21^ suggesting that peripheral nerves may be involved in the precise regulation of limb regeneration.

Regarding the mechanisms by which peripheral nerves regulate salamander limb regeneration, diffusible nerve factors were shown to cross the filter and promote blastema cell proliferation.^29^ In response to initial nerve injury signals, SCs undergo phenotypic changes, downregulating myelin proteins, such as Krox20, Sox10, and neuregulin 1. Similar to their progenitors, negative regulators of myelination and growth-promoting proteins, such as Notch and c-Jun, are upregulated in SCs. These changes facilitate the transition of SCs from typical peripheral glial cells to repair cells. Together with blastema-infiltrating nerve fibers, repair SCs release a variety of molecules in the microenvironment of the blastema. Although poorly understood, many diffusible nerve factors have been shown to regulate regenerative processes in the salamander, including substance P (SP),^30,31^ platelet-derived growth factor (PDGF),^32^ fibroblast growth factors, bone morphogenetic protein (BMP),^33,34^ glial growth factor,^35^ newt anterior gradient (nAG),^36^ transferrin,^37^ and neuregulin.^38,39^ These paracrine factors are produced by neurons or repair SCs and provide signals to immune cells or stem/precursor cells to support regeneration.^40^

**Fig. 1:**
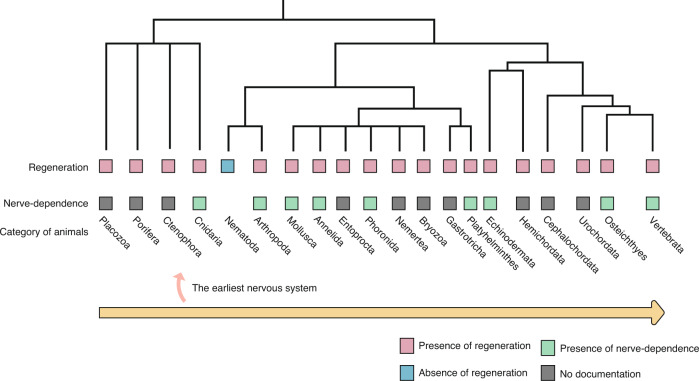
Distribution of regeneration and its nerve dependence in animals from lower to higher species. This phylogenetic tree topology contains almost all animal species. The yellow arrow at the bottom from left to right indicates the gradient from animals that emerged earlier in evolutionary history (lower organisms) to those that evolved later (higher organisms). The distribution of regeneration capacity in most animal phyla could be confirmed in at least one member. “Presence of regeneration” means that there is at least one verified taxon in the corresponding phylum that has the ability to regenerate complex parts of the body; it does not refer to all the taxa included. “Presence of nerve dependence” means that there is at least one well-substantiated report indicating the function of the nervous system in regeneration. “No documentation” means that more reliable studies are needed on the topic. Tree topologies and regeneration data are based on ref. ^292^ Data on nerve dependence are based on refs. ^21,22,41–45,293–296^

The participation of peripheral nerves in regeneration is not, however, limited to salamanders or amphibians but is found in many other species, from lower organisms such as sea anemones,^41^ hydras (*Cnidaria*),^42^ planarians (*Platyhelminthes*),^43^ and starfish (*Echinodermata*),^44^ to vertebral organisms such as zebrafish,^45^ which possess the capacity for regeneration under neural effects (Fig. 1). Notably, the existence of species such as *Placozoa* and *Porifera*, which have the ability to regenerate without the nervous system, is coincident with the observation of the aneurogenic limb, which regenerates without a nerve supply but develops nerve-dependent regeneration after nerve transplantation and innervation.^22^ New findings in mammals are consistent with previous studies. For example, MRL/MpJ mice can regenerate injured ear tissue through blastema-like structures, while denervation inhibits the formation of the blastema.^46^ Furthermore, in murine digit tip regeneration, SCs in injured nerves have been shown to dedifferentiate and promote blastema proliferation in a paracrine manner.^47^ The process from blastema formation to appendage regrowth also requires complex regulation, such as cell pattern and polarity,^48^ which depends on the location (distal or proximal, dorsal or ventral) of cells within the newly formed tissue. Patterning defects have also been noted in denervated and regenerating murine digits.^17^ Taken together, these results indicate the widespread participation of peripheral nerves in mammalian tissue regeneration.

Advances in the understanding of tissue regeneration in other species have shed light on regeneration mechanisms in mammals. nAG, which is mainly produced by repair SCs during salamander limb regeneration, binds to its receptor Prod1 to stimulate blastema cells to enter the S phase of the cell cycle and promote blastema expansion.^36^ The administration of nAG rescues 50% of the effect of denervation on salamander limb regeneration.^36^ Unfortunately, although it is heralded as the key to the field of regenerative medicine, nAG has no orthologous protein in humans.^49^ The closest proteins in humans are anterior gradient protein 2 (AGR2) and AGR3, but their potential role in regeneration remains unclear, as these factors lack the features of secreted proteins.^49^ Nevertheless, cells in the blastema were once considered to be multipotent and homogeneous based on studies of salamander regeneration.^50^ Genetic lineage tracing and single-cell transcriptomic profiling of mammalian digit regeneration, however, have shown that the heterogeneous blastema consists of many cell types.^51,52^ Later studies indicated that the origin cells of the blastema were developmental lineage-restricted, which is prevented across germline lineages^51,53^ but is relatively flexible in the consequent generated mesenchymal lineage,^54^ suggesting that the newly formed tissue differentiated from blastema cells depending on their respective origin and regenerative microenvironment. Distinct from the paracrine pathway, most endoneurial mesenchymal cells in peripheral nerves have multipotential differentiation abilities, as shown by upregulated expression of genes such as *Aldh1a2*, *Col11a1*, *Cthrc1*, *Inhbb*, *Kng2*, and *Wif1* and downregulated expression of genes related to connective tissue after nerve injury. These neural crest-derived cells (NCCs) not only contribute to blastema formation but also subsequently differentiate into dermis or bone in response to specific environmental cues.^55^

Overall, studies on both mammals and phylogenetically distant animals, such as amphibians, show how peripheral nerves can regulate regeneration: (1) the secretion of neuropeptides, neurotransmitters, and other neural molecules^21,56^ and (2) the differentiation of stem/precursor-like cells and/or transdifferentiation from endoneurial mesenchymal cells in injured peripheral nerves.^5^

## Distribution of peripheral nerves in the skeletal system

The afferent nerves of the peripheral system are collectively known as sensory nerves. The efferent nerves consist of motor nerves and autonomic nerves, which can be further categorized into the sympathetic nervous system (SNS) and the parasympathetic nervous system (PSNS) (Fig. S1). Sensory nerves extend from their cell bodies in the dorsal root ganglia (DRGs) of the spinal cord, and cranial bone is innervated by sensory nerves emanating from cranial nerve ganglia, such as the trigeminal ganglion. Depending on their diameter and myelination, sensory nerves can also be classified into thin, nonmyelinated C fibers and myelinated A fibers.^57,58^ It has been shown that pain after bone fracture is mainly detected by Aδ fibers and C fibers^59^ because Aδ fibers and C fibers account for almost all sensory nerves that innervate bone.^60–62^ C fibers can be further divided into peptidergic and nonpeptidergic fibers, which can transduce noxious chemical and thermal stimulation.^57^ Postganglionic fibers of the ANS are similar to C fibers, as they are thin and nonmyelinated.^63^

**Fig. 2:**
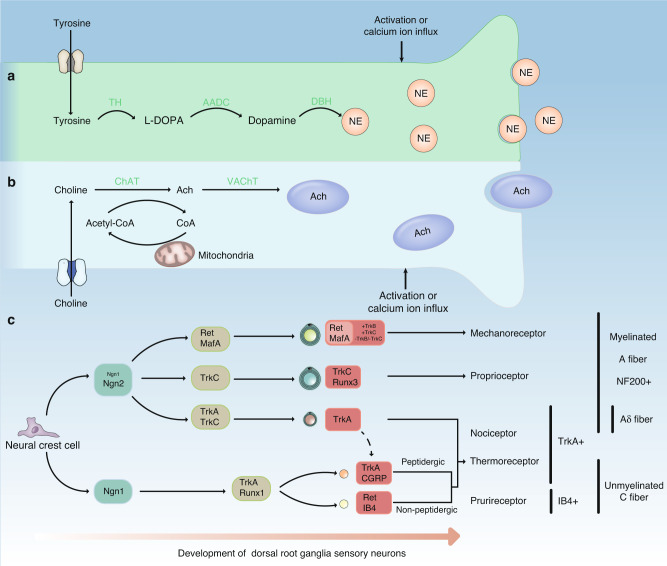
Main biomarkers of the ANS and sensory nerves. **a** Norepinephrine (NE) is synthesized from tyrosine by a multienzyme pathway. Tyrosine hydroxylase (TH) converts tyrosine to L-DOPA. L-Aromatic amino acid decarboxylase (AADC) converts L-DOPA into dopamine, which is finally hydroxylated by DA-β-hydroxylase (DBH) to produce NE. **b** Acetylcholine (ACh) is synthesized from choline and acetyl coenzyme A (acetyl-CoA) with catalysis by choline acetyltransferase (ChAT), and then vesicular ACh transporter (VAChT) stores ACh in vesicles. **c** Development of DRG sensory neurons. All DRG sensory neurons develop from neural crest cells and gradually differentiate into different lineages with sophisticated regulation, which is not fully understood. Finally, after progressive diversification, these immature neurons develop into various sensory neurons that transduce different kinds of sensation, including mechanoreceptors, proprioceptors, nociceptors, thermoreceptors and pruriceptors (sensitive to histamine-independent itch). The length of short vertical lines marked with specific biomarkers on the right corresponds to the different lineages on the left. The most representative expression of this lineage during development is marked in boxes. The horizontal axis shows the relative development of DRG neurons, which means that Ngn1^+^ neurons are late developers. Dotted lines indicate deductions. Ngn1 Neurogenin-1, Ngn2 Neurogenin-2, TrkA Tropomyosin receptor kinase A, NF200 Neurofilament 200, IB4 Isolectin B4

Peripheral innervation in the skeletal system has gradually been outlined using immunoreactivity to biomarkers of anabolic processes of postganglionic representative neurotransmitters (Fig. 2a, b). The distribution of the SNS in bones can be visualized through norepinephrine (NE), which is synthesized from the amino acid tyrosine by two important enzymes: tyrosine hydroxylase (TH) and dopamine β-hydroxylase.^64,65^ Neuropeptide Y (NPY) is mainly released by sympathetic terminals and accompanied in the periphery by NE,^66^ which is associated with NPY on SNS visualization. The location of acetylcholine (ACh) in the vesicular ACh transporter (VAChT) and choline acetyltransferase (ChAT) allows mapping of PSNS distribution.^67,68^ Major sensory neurotransmitters, such as calcitonin gene-related peptide (CGRP) and SP, can be used to identify peptidergic sensory nerves because of their relatively exclusive origins.^69–72^ Other primary molecules of sensory nerves, such as tropomyosin receptor kinase A (TrkA), neurofilament 200, and isolectin B4, are biomarkers of different sensory lineages (Fig. 2c).^73–75^ It has been reported that the proportions of CGRP^+^ peptidergic sensory axons and TH^+^ sympathetic adrenergic axons in the total nerve population innervating the skeleton were at least 20%–30% and 25%–50%, respectively.^76^ Reliable characterization of non‐peptidergic sensory axons that innervate bone is needed.

The presence of the SNS and sensory nerves in the skeletal system has been visualized by immunolabeling techniques.^61,71,72,77,78^ However, since a group of postganglionic sympathetic neurons exhibits a cholinergic phenotype in bone,^79,80^ the accuracy of innervation of the PSNS in the bone as delineated by positive VAChT and ChAT immunoreactivity is compromised. Ingeniously, injection of a recombinant pseudorabies virus into the distal femoral metaphysis labels the intermediolateral column at the thoracic level with SNS innervation, as well as the intermediolateral column at the sacral spinal cord segment where PSNS preganglionic neurons are located, which establishes a strong connection between PSNS and bone innervation.^81^ Direct evidence tracing the autonomic postganglionic nerves in the bone to parasympathetic ganglia is expected to provide further support for their relationship.

The involvement of the PNS with the skeleton has been reported as early as embryonic development. Mesenchymal condensation directly differentiates into bone via intramembranous ossification during embryonic development to form flat bones, whereas during endochondral ossification, cartilaginous tissues form and are then replaced by mineralized bone.^82^ During the embryonic development of long bones in mice, endochondral ossification begins on approximately embryonic day 15 (E15), and secondary ossification occurs on approximately postnatal day 5 (P5).^13,83^ TrkA^+^ sensory nerves innervate the developing femur at the perichondrial region adjacent to sites of primary ossification on E14.5 and are present at the epiphyseal surface of the femur on P0.^13^ Nerve growth factor (NGF), which supports neuronal survival and guides axonal growth, is expressed in perichondrial cells as early as E14.5.^13^ The requirement of TrkA signaling in sensory nerves for the formation of primary and secondary ossification is further suggested by the reductions in innervation, angiogenesis, and osteogenesis resulting from the disruption of NGF-TrkA signaling.^13^ After birth, the density of nerves continues to increase in growing bones, but NPY ^+^ nerve fibers could not be detected until P4.^84^ In addition to long bone development, innervation also participates in osteogenesis through intramembranous ossification. The mandibular branch of the trigeminal nerve develops preferentially in the primordium of the lower jaw,^85^ and the ossification center of the mandible is just adjacent to the nerve bundle, extending along the inferior alveolar nerve.^86^ Genetic disruption of TrkA signaling in sensory nerves leads to early closure of cranial sutures.^87^

Generally, peripheral nerves are thought to accompany blood vessels,^88^ and this holds true in mature bones. Sensory nerves, as well as the SNS, parallel the vascular structures to reach bone as a mixture of motor, sensory and sympathetic nerves.^89^ The major nerves consist of mixed neural components innervating the periosteum in a meshwork pattern,^71^ particularly the inner cambium layer, which possesses a high cell density consisting mainly of periosteum-derived MSCs, osteoclasts and osteoblasts.^90,91^ Through nutrient canals, vertical Haversian canals, and transverse Volkmann’s canals, sensory and sympathetic nerve fibers penetrate into the cortical bone parallel to the vasculature and then into the bone marrow.^92–94^ Rodent studies have demonstrated that the periosteum has the highest density of innervation, followed by the bone marrow and mineralized cortical bone.^76,77,92^ Although there is a possibility that rodents and humans share a similar innervation pattern,^95,96^ consistent innervation density of the bone in humans has not been shown until recently.^97^ The significant predominance of CGRP^+^ nerves relative to TH^+^ nerves in the periosteum is reversed in bone marrow,^76^ indicating the different roles/mechanisms of sensory nerves and the SNS in regulating bone hemostasis and regeneration. When running parallel with the vasculature, the sensory nerves and SNS tend to run linearly or spirally around vessels, respectively.^92,98^ The distribution of peripheral nerves in bone constitutes the foundation of PNS-mediated regulation of the skeletal system. PSNS have been shown to exist in bone,^81^ but detailed and quantitative experiments are still lacking. Furthermore, the same subcompartment may exhibit heterogeneity in innervation when there is active metabolism, which is associated with more innervation, such as in epiphyseal trabecular bone.^77^ The physiological innervation of the PNS in different compartments of bone begins during embryonic development, facilitates the regulatory potential of peripheral nerves, and participates in various physiological or pathological processes in bone.

## Distribution of peripheral nerves in the skeletal system following the fracture

Following injury, molecular and cellular changes are observed in the neuronal body, cells resident in peripheral nerves, and at the site of injury. These changes in peripheral nerves after bone fracture are prerequisites for the initiation of bone regeneration.

### Changes in peripheral nerves after fracture

Bone fractures are common injuries caused by external forces or pathological changes that weaken the bone structure, and the PNS responds actively to the damage signal.^99^ The PNS interrupts synapses and switches to a regenerative state,^100–102^ reducing the production of neuropeptides for regeneration-associated metabolism, as shown by the downregulation of synthesis in the perikarya.^103–106^ However, neuropeptides, such as CGRP and SP, are thought to be increased in the fracture region.^107,108^ Apart from the fact that activation of the PNS directly contributes to the release of neurotransmitters,^109^ synthesis in injured axons (axonal synthesis) rather than the perikarya during regeneration partly accounts for the increase in neurotransmitters.^110^ When peripheral nerves are injured at a bone fracture site, injury signals travel in a retrograde manner along the proximal axon to the cell bodies to initiate regeneration.^111^ The distal axon undergoes Wallerian degeneration, in which the axon degenerates, myelin breaks down, the blood–nerve barrier is permeabilized, and the resultant myelin debris containing axonal growth inhibitory signals is cleared first by SCs and later by recruited macrophages.^111,112^ The roles of macrophages in peripheral nerve regeneration have been exhaustively reviewed recently.^113^ Other immune cells, such as mast cells, neutrophils, and T cells, are also recruited to the injured site as well as the distal stump and are involved in pain induction, but their role in nerve regeneration is still obscure.^114^ Regenerating proximal axons extend to the target organ following the guidance of SC basal lamina tubes, which are provisional channels formed by the proliferation of repair SCs.^111^ Delayed reinnervation of the target organ results in regeneration failure because of the degeneration of SC tubes.^111^

The mechanical deformation associated with bone fractures or defects activates the Aδ or C fibers, which transmit initial pain stimuli to the relevant cortical areas,^115^ and then central signals descend to the fracture site, resulting in local regulation such as the release of catecholamine by sympathetic arousal. Inflammation at the fracture site sensitizes the sensory nerves, which lowers the response threshold to noxious mechanical, chemical and thermal stimuli.^109,116–118^ There is a wide range of receptors at the terminals of sensory nerves that detect specific inflammatory mediators and growth factors,^119^ and the activation of these receptors triggers a series of downstream changes, such as the phosphorylation, gating, and upregulation of ion channels (e.g., Nav1.7, Nav1.8, Nav1.9, TRPV1, and TRPA1),^109^ leading to sensitization as well as further neurotransmitter release.^109,120^ Cytokines (histamine, TNF, IL-1β, IL-6, IL-17A), lipid mediators (prostaglandin E2 (PGE2), leukotriene B4), and growth factors (NGF, brain-derived neurotrophic factor (BDNF)) are produced mainly by mast cells, neutrophils, macrophages, and Th17 or γδT cells and contribute substantially to the sensitization of sensory nerves.^121^ For example, the binding of TNFα and its receptor (TNFα receptor 1, TNFR1) on the terminals phosphorylates Nav1.8 channels to facilitate channel opening.^122^ In short, Wallerian degeneration and inflammation are the primary responses of peripheral nerves during bone fracture.

### Changes in peripheral nerves during bone repair and regeneration

Bone regeneration completely restores the original microarchitecture and is accompanied by reinnervation. These two seemingly independent processes are tightly intertwined in reality. Using growth-associated protein 43,^123^ which is more prevalent in differentiating and regenerating neurons than in mature neurons,^124^ or Thy-1,^110^ a pan-neural gene,^125^ the changes in peripheral nerves during reinnervation at the fracture site can be clearly observed. The reinnervation process precedes angiogenesis at the early stage of bone repair/regeneration.^126,127^ Previous studies have reported that ectopic sprouting of sensory and sympathetic nerve fibers after bone trauma greatly contributes to the hyperinnervation of all the compartments at the fracture site.^123,127–129^ Moreover, researchers concluded that an adequate density of innervation was the prerequisite for initiating regeneration in salamanders,^22,130^ indicating that hyperinnervation after bone fracture may be required for regeneration. During callus formation and maturation, peripheral nerves sprout while the bone matrix is deposited, gradually reduced, and finally restricted to the outer fibrous capsule of the hard callus when injured nerves are trimmed.^123^ Both CGRP^+^ and TH^+^ spouting nerve fibers can participate in reinnervation, and CGRP^+^ nerve fibers contribute the most.^123,129,131^ Reinnervation of bone has also been confirmed by findings in calvarial bone defect regeneration.^132^ After bone repair, the PNS fibers in bone typically return to physiological levels. However, in fracture nonunion, hyperinnervation remains around the bone, periosteum, cortical bone, and bone marrow.^128^ At present, the remaining hyperinnervation is viewed as a pathological state associated with chronic pain.^133^ These observations suggest an interdependent relationship between bone regeneration and PNS regeneration after bone fracture.

## Peripheral nerve regulation of bone regeneration

Physiological innervation of different compartments and the intertwined schedule of regeneration make the PNS a strong candidate for the regulation of bone regeneration. With continuous research on the role of the PNS in bone regeneration, the emerging importance of the PNS echoes the well-known role of nerves in limb regeneration. Early relevant denervation experiments provided insight into this phenomenon. Sciatic nerve resection results in defective callus formation in rats and rabbits.^25^ Inferior alveolar denervation impairs regeneration of the mandibular bone defect in rats.^26^ The administration of high-dose capsaicin to destroy capsaicin-sensitive sensory nerves decreases Mg^2+^-mediated promotion of fracture healing.^108^ Similarly, disruption of TrkA signaling blunts angiogenesis and delays callus formation in mice.^124^ Knockout of GGRP in mice inhibits bone healing.^134^ Sympathectomized mice have delayed cartilaginous callus formation and callus mineralization.^135,136^ The findings of these in vitro and animal experiments are compatible with clinical observations of bone development. Congenital insensitivity to pain with anhidrosis, which is a hereditary neurodevelopmental disorder caused by mutations in *TRKA*,^134^ is associated with short stature and delayed fracture healing.^137^ In regard to unsuccessful bone regeneration, initial injury of the nerve or vasculature may be associated with a secondary operation after nonunion repair.^136^ The nonhealing area in spondylolisthesis patients has been shown to coincide with the region lacking innervation.^138^ In addition, postoperative absorption has been a challenge in the use of vascularized iliac bone to reconstruct the jaw,^139^ and 10 out of 22 patients who were treated with neurorrhaphy between the ilioinguinal nerve and the inferior alveolar nerve or auricular nerve during the reconstruction of the mandibular bone exhibited reduced bone absorption.^140^ These clinical observations indicate the involvement of peripheral nerves in regulating osteogenesis from the embryonic phase to adulthood.

**Fig. 3:**
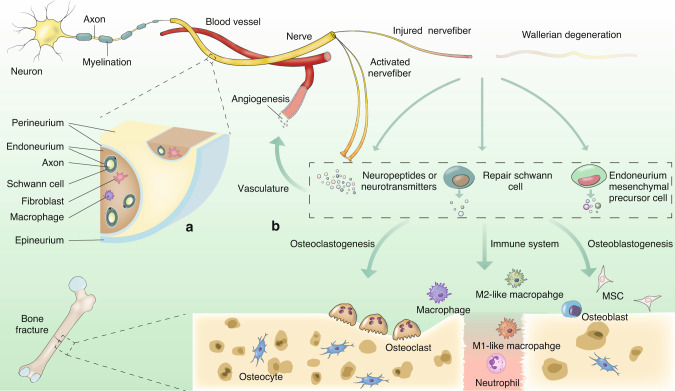
Illustration of possible ways in which the PNS can regulate bone regeneration following trauma. **a** Structure of peripheral nerves and nerve-resident cells involved in bone regeneration. **b** The PNS regulates bone regeneration in two main ways: neuropeptides or neurotransmitters and nerve-resident cells. Neuropeptides or neurotransmitters can be secreted by injured and activated nerve fibers. Nerve-resident cells, mainly SCs and endoneurial mesenchymal cells, alter their transcription and translation, changing their ordinary phenotype to a reparative one, which is similar to their precursor cells (these cells have a common origin from NCCs) after nerve injury. Repair SCs can secrete regulatory molecules, such as oncostatin M and PDGF-AA, to promote regeneration, and endoneurial mesenchymal cells in injured nerves differentiate into bone-lineage cells. The development of inflammation, the invasion of newly formed vessels, the differentiation of osteogenesis and osteoclastogenesis are all regulated by these responses of the PNS after bone trauma

The discovery of relevant neuropeptides and their receptors in bone-lineage cells is a cornerstone of their crosstalk with the PNS (Table 1), but how are the regulatory signals transported from neurons to the bone? Although osteocytes, osteoblasts, osteoclasts, and vascular endothelial cells are located in close proximity to free nerve endings,^98,141,142^ the rarity of these direct connections casts doubt on the hypothesis of synaptic connections, which has not yet been found.^141,143,144^ It is uncertain whether the effects of PNS fibers on bone regeneration occur in a direct or indirect manner or both. Currently, two main ways whereby the PNS regulates bone regeneration have been proposed, as shown in Fig. 3.

**Table 1:** Expression of receptors involved in PNS-mediated regulation of bone regeneration in corresponding bone cell lineages

Molecule	Receptors	Bone cell lineage	Action
α-CGRP	CRLR, RAMP1	Rat PDSCs	Promote osteogenic differentiation^107^
		Rat BMSCs	Enhance osteogenic differentiation^149,150^
			Enhance proliferation^149^
		Mouse BMSCs	Promote osteogenic differentiation^152^
		Mouse osteoblast precursors	Promote osteogenic differentiation^148^
		Human osteoblasts	Decrease apoptosis of osteoblasts^151^
			Decrease OPG secretion^154^
SP	NK‐1R	Rat BMSCs	Promote BMP-2 and VEGF expression and induces osteoblastic differentiation^161^
			Promote migration^161,163^
		Rat osteoblasts	Increase the ratio of RANKL to OPG expression^156^
			Induce proliferation^156^
		Mouse BMSCs	Promote BMSCs proliferation and osteogenic differentiation^158^
		Mouse osteoblasts	Increase RANKL expression^158^
		Mouse BMMs	Activate NF-κB in BMMs^158^
		MC3T3-E1 cells	Enhance osteoblastic differentiation^162^
NE	α1-AR	Rat BMSCs	Stimulate proliferation^178^
		MC3T3-E1 cells	Stimulate osteoblastic proliferation^169^
	β2-AR	Mouse osteoblast progenitor cells	Increase RANKL secretion^173^
		Mouse osteoblasts	Reduce osteoblast proliferation^290^ Suppress osteoblast activity^144^ Inhibit osteoblasts differentiation^171^
		MLO-Y4 cells	Increase the ratio of RANKL to OPG^174^
		Human BMSCs	Reduce cell proliferation^174^
ACh	nAChRs	Mouse osteoblasts	Stimulate proliferation^81^
		Mouse osteoclasts	Promote apoptosis^81^
		Mouse BMMs	Inhibit osteoclastogenesis^185^
NPY	Y1	Rat BMSCs	Y1 antagonist promotes osteogenic differentiation of BMSCs^193^
		Mouse BMSCs	Inhibit BMSC differentiation^192^
		Mouse osteoblasts	Inhibit function of osteoblasts^190,192^
		Human BMSCs	Inhibition of Y1 receptor signaling enhances osteogenic differentiation^191^
VIP	VPAC1	Rat BMSCs	Promote osteogenic differentiation^197^
NGF	TrkA	Mouse chondrocytes	Promote ossification^210^
		Mouse osteoblasts	Promote differentiation^123^
		MC3T3-E1 cells	Stimulate differentiation^207^ Decrease apoptosis^208^
BDNF	TrkB	MC3T3-E1 cells	Promote migration^216^
		Human BMSCs	Promote osteogenic differentiation^215^
			Increase the level of RANKL^217^
NT-3	TrkC	Rat BMSCs	Promote osteogenic differentiation^206^
Sema3A	Plexin-A	Mouse osteoblasts	Promote differentiation^223,224^
		Mouse osteoclasts	Decrease differentiation^223,224^
		Mouse BMMs	Inhibit migration^224^
		Human BMSCs	Promote osteogenic differentiation^291^
Sema3E	Plexin-D1	Mouse BMMs	Decrease the formation of osteoclasts^227^
		Mouse osteoblasts	Inhibit migration^227^
Sema4D	Plexin-B1	Mouse osteoblasts	Inhibit differentiation and migration^228^
EphB2	Ephrin-B1	Mouse BMSCs	Knockout suppresses BMSCs differentiation^235^
		Mouse osteoblasts	Promote differentiation^231^
		Human BMSCs	Promote BMSCs migration and chondrogenic differentiation^232^
EphB4	Ephrin-B2	Mouse chondrocytes	Lack of Ephrin-B2 or EphB4 decreases osteoblastic differentiation of chondrocytes^233,236^
		Mouse osteoblasts	Lack of Ephrin-B2 or EphB4 decreases osteoblast differentiation^233,236^
		Kusa 4b10 cells	Promote osteoblast differentiation^234^

At least one in vivo study of bone regeneration is required before a molecule can be listed in the table

*CGRP* calcitonin gene-related polypeptide, *SP* substance P, *NE* norepinephrine, *ACh* acetylcholine, *NPY* neuropeptide Y, *VIP* vasoactive intestinal peptide, *NGF* nerve growth factor, *BDNF* brain-derived neurotrophic factor, *NT-3* neurotrophin-3, *Sema* Semaphorin, *Eph* erythropoietin-producing hepatocellular carcinoma, *PDSC* periosteum-derived stem cell, *BMSC* bone marrow mesenchymal stem cell, *BMM* bone marrow-derived macrophage

## Neuro-skeletal regulation during bone regeneration

**Fig. 4:**
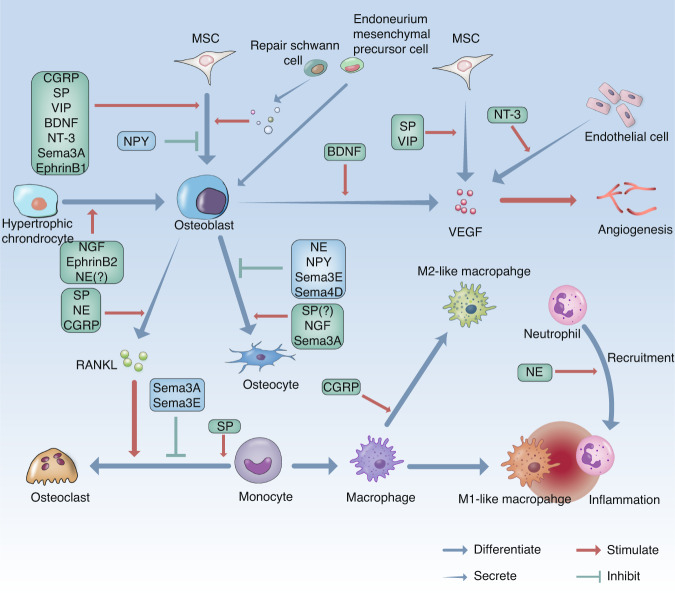
Illustration showing how PNS nerve fibers regulate bone regenerative processes following injury. The basic regeneration processes are represented by blue arrows. Neuropeptides or neurotransmitters positively or negatively regulate osteoblastic differentiation in MSCs, osteoclastogenesis in monocytes, tube formation by endothelial cells, type switching in macrophages and the recruitment of immune cells during the inflammatory phase of bone regeneration. Dedifferentiated SCs and endoneurium mesenchymal precursor cells contribute to new bone formation via direct osteoblastic differentiation or indirect secretion to stimulate the differentiation of MSCs. Red arrowheads represent a stimulatory effect, while green flatheads represent an inhibitory effect. Green boxes contain stimulative neuropeptides or neurotransmitters for the corresponding process, and blue boxes contain suppressive neuropeptides or neurotransmitters. “?” indicates that more reliable evidence is needed. CGRP calcitonin gene‐related peptide, SP substance P, NE norepinephrine, NPY neuropeptide Y, VIP vasoactive intestinal peptide, NGF nerve growth factor, BDNF brain-derived neurotrophic factor, NT-3 neurotrophin-3, Sema3A Semaphorin 3A, Sema3E Semaphorin3E, Sema4D Semaphorin4D

After bone trauma, nerve activation in bone and nerve regeneration at the injured site occur simultaneously. The upregulation of a wide variety of neuropeptides or neurotransmitters in activated sensory nerves and in the ANS is regulated by signals to the CNS and contributes to shaping the dynamic microenvironment of bone regeneration (Fig. 4). In addition, neurotrophins and axon guidance family proteins, which are highly active during nerve regeneration and are regulated by the PNS and other cells in the bone microenvironment, are also responsible for mediating bone regeneration. Cells in nerves, such as SCs and endoneurial mesenchymal cells, convert to a regenerative phenotype that is more similar to their precursor state. Through paracrine signaling or possible redifferentiation, these transcription-altered cells not only participate in nerve regeneration but also communicate with bone-lineage cells for bone regeneration.

### CGRP

CGRP, which is the major neurotransmitter of sensory nerves, is produced by alternative splicing of the CALC gene.^145,146^ CGRP is transcribed from different regions in the *CALC* gene and exists in two forms: αCGRP and βCGRP. βCGRP is mainly found in the enteric nervous system,^70,147^ which is consistent with the observation that αCGRP is the main cause of elevated levels of CGRP in serum after the fracture in mice.^148^ The CGRP receptor is a heterodimer of calcitonin receptor‐like receptor (CRLR), which is a G‐protein coupled receptor (GPCR), and its coreceptor, receptor activity‐modifying protein 1 (RAMP1).^70^ CGRP is increased in injured bone tissues, and the expression of its receptor also increases at the early stages of bone regeneration.^107,148^ The administration of CGRP promoted the migration of bone marrow mesenchymal stem cells (BMSCs) to the fracture site and osteogenic differentiation in rats.^149^ Disruption of CGRP signaling with receptor antagonists such as CGRP 8–37 and nonpeptide CGRP antagonists (BIBN4096BS) or with short interfering RNA significantly inhibited new bone formation.^107,149^ Further studies demonstrated that CGRP activated the cAMP/protein kinase A (PKA) signaling pathway and subsequently inhibited BMSC apoptosis and promoted BMSC proliferation and osteogenic differentiation by enhancing Wnt/β‐catenin in vitro.^150,151^ The overexpression of RAMP1 in BMSCs enhances the osteogenic differentiation of BMSCs, while blocking Yap1 blocks this effect, indicating that CGRP can promote BMSC differentiation via the Hippo/Yap pathway.^152^ Impairment of cartilaginous callus remodeling and callus bridging in *CGRP*^−/−^ mice is linked to the low expression of key mediators (adiponectin, adipocyte protein 2, and adipsin) of the PPARγ pathway,^134^ the inhibition of which impairs the osteogenesis effect of BMP-2.^153^ CGRP also has a negative effect on osteoclastogenesis by suppressing osteoprotegerin (OPG) production in osteoblasts.^154^ Preclinical use of electrical stimulation of lumbar DRGs can promote the release of CGRP and effectively enhance femoral fracture healing, providing an innovative strategy for further clinical use.^155^

### Substance P

SP is another major neurotransmitter that is released by activated sensory nerves and is usually released with CGRP.^153^ SP is encoded by the *tachykinin precursor 1 (TAC1)* gene and binds to its receptor neurokinin‐1 receptor (NK‐1R) to regulate target cells. NK-1R has been identified in BMSCs,^135^ osteoblasts and osteoclasts.^156–158^ SP^+^ nerve fibers are increased following a fracture or bone defect,^108,159^ as is the expression of SP in injured bone.^160^ SP has been reported to improve the osteoblastic differentiation of BMSCs and MC3T3-E1 cells by activating the Wnt/β-catenin signaling pathway.^161,162^ Furthermore, SP embedded on titanium substrates can also promote BMSC recruitment during bone healing.^163^ On the other hand, blocking NK-1R reduces the expression of osteocalcin and collagen 1A2 and 2A1 during bone regeneration, resulting in significantly impaired biomechanical strength.^164^

While having an impact on osteogenesis differentiation, SP also modulates osteoclastogenesis. SP can promote osteoclastogenesis independently of RANKL by inducing NF‐κB in osteoclast precursors^165^ or by stimulating osteoblast lineage cells to produce RANKL.^158^ This effect has been reported to be dose dependent: SP promotes osteoblast differentiation and matrix mineralization when the concentration is greater than 10^−8^ mol·L^−1^ and suppresses these processes at less than 10^−8^ mol·L^−1^.^135^ Investigation of the regenerative process of *TCA1*^−/−^ mice provided new evidence. Compared to bone regeneration in WT mice, *TAC1*^−/−^ mice showed a decrease in the total area of cartilaginous soft callus tissue, reduced numbers of osteoclasts and osteoblasts at the fracture site, and impaired resistance to torsional failure load.^135^ Furthermore, recent observations in ovariectomized *TAC1*^−/−^ mice contradict previously published results with regard to the hypertrophic chondrocyte area, but the number of osteoclasts in the fracture site was consistent with the final outcome.^166^ Whether these discrepancies are the result of ovariectomy is not yet clear.

### Norepinephrine

NE is an important neurotransmitter that is released by noradrenergic nerves, which are mainly postganglionic fibers of the SNS. There are two forms of the receptor, the α‐adrenergic (α‐AR) and β‐adrenergic receptors (β‐AR), each containing many subtypes.^89^ These receptors have been observed in various bone-lineage cells.^144,167–170^ High sympathetic tone increases epinephrine, which can be detected in urine, resulting in the suppression of osteoblast activity, as evidenced by abnormal morphology and reduced Ki67 expression in osterix^+^ cells.^171^ A β_2_-AR antagonist has been shown to rescue these changes, highlighting the negative effect of NE on osteoblastic differentiation.^171^ NE may also inhibit the proliferation of hBMSCs through β_2_-AR-induced phosphorylation of ERK1/2 and PKA.^172^ In addition, NE induces osteoclastogenesis by activating RANKL/OPG.^173,174^ The nonselective β‐AR blocker propranolol has been shown to enhance bone healing in rats;^175^ interestingly, administration of propranolol to posttraumatic stress disorder (PTSD) mice with femur fractures ameliorated the defect in new bone formation.^176^ However, given that the effect of nonselective β-AR blockade differs greatly between mice and humans in the context of bone metabolism,^177^ further evidence on the effects of NE and β‐AR on human bone regeneration is still needed. For α‐AR, DNA synthesis in BMSCs is reported to increase in rats via α_1_-AR.^178^ The administration of phenylephrine, a nonspecific α1-AR agonist, promotes the proliferation of MC3T3-E1 cells by increasing the expression of the transcription factor CCAAT/enhancer-binding protein δ (Cebpd).^169^ Its detailed role in bone regeneration requires further elucidation.

Significantly, α- and β-ARs are GPCRs, and downstream binding to α-AR decreases cAMP and subsequently inhibits PKA, while binding to β-AR induces the opposite effects. However, the binding affinity of NE largely depends on its concentration:^179^ at concentrations less than 10^−8^ mol·L^−1^, NE preferentially binds to α‐AR, while at concentrations higher than 10^−6^ mol·L^−1^, β‐AR is preferred. The concentration of NE in bone marrow ranges from 10^−9^ mol·L^−1^ (physiological) to 10^−5^ mol·L^−1^ (pathological).^179,180^ Therefore, the effect of NE during the process of bone regeneration may vary dynamically depending on the stage of the regenerative process.

### Acetylcholine

Identifying the involvement of PSNS in the skeletal system draws attention to the role of ACh in bone regeneration. As previously described (Fig. 2b), ACh can function as a transmitter in the PSNS by binding to nicotinic (nAChRs) and muscarinic acetylcholine receptors (mAChRs),^68^ both of which have been found in bone-lineage cells.^181–183^ Past studies demonstrated that ACh promoted osteoblastic proliferation^81,182^ but had little effect on osteoblastic differentiation.^81^ Unexpected negative effects on alkaline phosphatase (ALP) activity in osteoblasts have also been reported.^181^ An increase in bone resorption was observed in *α*_*2*_*nAChR*^−/−^mice through increased osteoclast numbers, and nAChR agonist administration increased apoptosis.^81^ Furthermore, RANKL-induced Ca^2+^ oscillation, the well-established osteoclastogenesis process,^184^ is inhibited by activation of nAChR, and subsequent weakened Ca^2+^-NFATc1 signaling leads to a negative regulatory effect on osteoclastogenesis.^185^ Donepezil, an acetylcholinesterase inhibitor (AChEI) that suppresses ACh degradation and increases the concentration of ACh, impairs bone healing by decreasing immune cell infiltration during the inflammation phase and reducing new bone formation.^186^ In a retrospective cohort study on Alzheimer’s disease patients with hip fracture, AChEI users had better radiographically observed union at the fracture site; better bone quality; and fewer healing complications, such as infection and delayed healing, than nonusers.^187^ Notably, the specific functions of the mAChR subtypes in the skeletal system should be clarified.^81,182,188^ Moreover, the M_3_ muscarinic acetylcholine receptor (M_3_AChR) in nerves plays an important role in bone metabolism.^188^

### Neuropeptide Y and vasoactive intestinal peptide

The receptors for NPY are GPCRs, and according to a rodent study, two of the five types (Y1 and Y2) are associated with the regulation of the skeletal system.^189^ Y1 has been observed in osteoblasts, while Y2 is located in the brain.^190^ Previous studies have demonstrated that NPY decreases cAMP levels in osteoblasts, impairing mineralization,^190^ while the administration of a Y1 antagonist promotes BMSC osteoblastic differentiation.^191^ Similarly, BMSCs isolated from Y1-silenced mice exhibited increased ALP activity and calcium nodule formation with increased expression of COL1, OCN, and Runx2, further illustrating the effect of NPY on the proliferation and apoptosis of BMSCs.^192^ The use of the Y1 receptor antagonist PD160170 on the femur improved the healing of bone defects.^193^ Recently, osteocytes from aging mice were shown to secrete NPY at high levels to induce adipogenesis in BMSCs, which is consistent with previous investigations.^194^

Vasoactive intestinal peptide (VIP), which is produced by enteric neurons, is also released by other peripheral nerves^195,196^ and is mediated by three types of GPCRs (VPAC1, VPAC2, PAC1).^196^ VIP can promote BMSC osteogenic differentiation through the Wnt/β‐catenin signaling pathway in rats.^197^ Although reported to inhibit BMSC proliferation,^198^ VIP-containing MeHA hydrogels increase the expression of vascular endothelial growth factor (VEGF), ultimately improving the healing of rat skull defects.^197^ Similarly, impaired bone regeneration after chemical sympathectomy can be rescued with VIP treatment, resulting in an increase in mineralized callus and improved callus bridging.^199^

### Neurotrophins

Neurotrophins are crucial for neuronal development and normal function and are involved in the formation of almost all neural circuits.^200^ Four types of neurotrophins have been discovered in mammals: NGF, BDNF, neurotrophin-3 (NT-3), and neurotrophin-4/5 (NT-4/5). All types can bind to the low-affinity receptor p75^NTR^ and to the specific high-affinity tropomyosin-related kinase (TRK).^200,201^ These factors function mainly through TRK; NGF binds to TrkA, BDNF and NT-4/5 bind to TrkB, and NT-3 binds to TrkC.^201^ Neurotrophins have been shown to be involved in bone regeneration through relevant receptors in recent years, especially NGF, BDNF, and NT-3.^202–206^

NGF is upregulated at the very early postfracture stage.^123^ In vitro, NGF can promote osteoblastic differentiation^207^ and has an antiapoptotic effect on MC3T3-E1 cells.^208^ A recent study demonstrated that most cells located in the periosteal callus, mainly periosteal/stromal cells and macrophages, were NGF^+^ cells during callus ossification, and the number of cells decreased during mineralization,^123^ which is consistent with the reinnervation of the injured PNS as previously described. Chemical disruption of NGF-TrkA signaling impairs the regeneration of the PNS and bone by reducing osteoblast activity and delaying callus mineralization.^123^ The administration of exogenous NGF improved healing in rabbit mandible fractures by increasing BMP-9 and VEGF levels.^209^ Similarly, entochondrostosis is enhanced by β-NGF supplementation, as evidenced by the increased expression of marker genes, such as *Ihh*, *Alpl*, and *Sdf-1*, which are associated with endochondral ossification. Local injection of NGF consequently promotes bone regeneration with deceased cartilage and increased bone volume.^210^ However, there are also controversial results suggesting that blockade NGF or TrkA by neutralizing antibodies reduces fracture-induced pain but has no side effect on bone healing as changes on biomechanical properties and callus formation and maturation are not observed in mice.^211,212^ A recent study showed that NGF has been used to treat traumatic nonunion in clinical trials, showing encouraging outcomes in promoting callus formation and fracture healing.^213^ A high-quality clinical trial with a large sample size is still needed to verify the effect of anti-NGF agents on bone pain and regeneration.

BDNF, which is critical in the development of the nervous system,^201^ was found in the brain after the discovery of NGF. BDNF also has an effect on the regulation of bone regeneration and can be released by inflammation-activated TrkA^+^ nerve fibers after bone trauma.^214^ BDNF can promote the proliferation and differentiation of hBMSCs.^215^ The promotion of bone regeneration by BDNF is achieved through the upregulation of integrin β_1_ via TrkB‐mediated ERK1/2 and AKT signaling.^216^ BDNF also enhances the production of RANKL by hBMSCs, contributing to osteoclastogenesis.^217^ These seemingly conflicting effects on bone may be due to the epigenetic regulation of BDNF transcription, whereby different physiological or pathological conditions induce alternative splicing and polyadenylation.^201^

The role of NT-3 in bone regeneration has been recently noted. The upregulated expression of NT-3 and its receptor TrkC has been verified during bone regeneration.^206^ By enhancing the expression of BMP-2 through Erk1/2 and Akt phosphorylation, NT-3 promotes osteogenesis in rat bone marrow stromal cells in vitro.^206^ Treatment with NT-3 during tibial fracture regeneration in rats promoted the expression of BMP-2 and TGF-β_1_, resulting in increased maximum load capacities.^218^ Systemic administration of NT-3 reduced the bone volume at the defects through immunoneutralization.^206^ It has been demonstrated that NT-4/5 is involved in pulp cell differentiation and regulating the function of periodontal ligament cells,^219,220^ but the association of NT-4/5 with bone regeneration remains unclear.

### Axon guidance family proteins

Axon guidance family proteins were initially identified during the development of the nervous system and provide attractive or repulsive cues to nerves during development or regeneration. Axon guidance family proteins guide the nerve to reach the correct target.^221,222^ Different from the previously described molecules, many members of this family and their ligands are membrane-bound. Class 3 semaphorins, a secreted class of axon guidance proteins, have long been linked to bone regulation. Semaphorin 3A (Sema3A), the receptor Nrp1, and the coreceptor Plxna1–3 have been found in bone tissue. Sema3A derived from sensory nerves has independent effect on bone by expediting innervation of sensory nerves and Sema3A promotes osteoblastic differentiation, enhances new bone formation, and inhibits RANKL-induced osteoclastogenesis via the Rac1 and Wnt/β-catenin pathways.^223,224^ The administration of Sema3A improved the formation of new bone in a similar way during calvarial defect healing in rats.^225^ The promotion of tibial fracture healing by Sema3A has also been observed in osteoporotic rats.^226^ Other Semaphorin 3 proteins also have effects on bone. Sema3E may inhibit the migration of osteoblasts and suppress the osteoclastogenesis of bone marrow-derived macrophages (BMMs).^227^ Furthermore, Sema4D has been demonstrated to exhibit negative effects on osteogenesis. By binding to the receptor Plexin-B1 on osteoblasts, osteoclast-derived Sema4D inhibits insulin-like growth factor-1 signaling, as well as migration, and consequently impairs bone formation.^228^ Disrupting Sema4D signaling increases the regeneration of defects in osteoporotic mice.^229^

The remaining classical axon guidance family proteins include ephrins, slits, and netrins. Erythropoietin-producing hepatocellular carcinoma (Eph) receptor tyrosine kinases and their ligands, ephrins, are important in bone formation. When Eph receptors on nerves mediate signal transduction, ephrins can also elicit a reverse signal in ephrin-expressing cells,^230^ such as bone-lineage cells (Table 1).^231–234^ Knocking out ephrin-B1 or -B2 in osteogenic progenitors significantly inhibited fracture healing.^235,236^ SLIT3 can promote the proliferation and migration of osteoblasts while suppressing the maturation of osteoclasts.^237^ Netrin-1 is involved in osteoclast differentiation,^238^ and as a part of bone-lineage cells that coordinates the PNS, osteoclasts can produce netrin-1 to guide the PNS.^239^ Studies on the effects of slits and netrins on bone-lineage cells during bone regeneration are still lacking. Moreover, a large number of axon guidance family proteins are theoretically needed to assist nerve growth, but very few new members have been discovered, such as draxin and phosphatidyl-β-D-glucoside.^222^

### PGE2 signaling

**Fig. 5:**
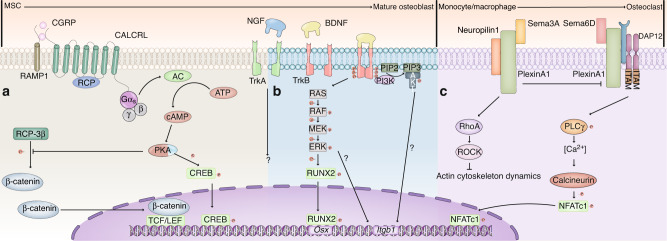
The diagram shows the possible signaling pathways of three representative neuropeptides (CGRP, BDNF, and Sema3A) that regulate bone formation. **a** Calcitonin gene‐related peptide (CGRP) is the main neuropeptide secreted by sensory nerves whose receptor is a G‐protein coupled receptor (GPCR). CGRP binds to the RAMP1-CALCRL complex and activates coupled Gαs subunits, which elevates the intracellular cAMP concentration and subsequently activates PKA. PKA inhibits the phosphorylation of β-catenin by GSK-3β. Unphosphorylated β-catenin translocates into the nucleus, where it associates with TCF/LEF transcription factors. Meanwhile, PKA activates CREB, which translocates into the nucleus and forms a homodimer or heterodimer that binds to the target gene. **b** Brain-derived neurotrophic factor (BDNF) is an important member of the neurotrophin family. BDNF binding to TrkB leads to the autophosphorylation of TrkB, which activates the PI3K/AKT and MEK/ERK pathways. RUNX2 is activated by the latter signaling pathway and upregulates the downstream molecule osterix to promote bone formation. The activation of ERK or AKT may contribute to the increased expression of integrin-β1, which is associated with migration. **c** Sema3A inhibits osteoclastogenesis in macrophages. Semaphorin 3A (Sema3A) is a secreted protein in the axon guidance protein family. Sema3A binds to its receptor PlxnA1–Nrp1 to inhibit the formation of the PlxnA1–TREM2–DAP12 complex, which can respond to ligands, such as Sema6D, to dephosphorylate NFATc1, thus inducing the transcription of osteoclast-specific genes. On the other hand, Sema3A activates RhoA, inhibiting BMM migration to prevent osteoclastogenesis. “?” indicates that more direct evidence is needed

The initial inflammatory phase drives bone regeneration, during which inflammatory mediators are produced to initiate a cascade of bone repair. PGE2 is a lipid mediator that is a member of the prostaglandin family. The key enzymes associated with PGE2 biosynthesis are prostaglandin E2 synthase-1 (mPGES-1) and COX. Inflammation-induced COX-2 expression contributes most to the catalysis of arachidonic acid into PGE2.^240^ PGE2 functions by binding with the receptors EP1–4, which are GPCRs that activate downstream effectors.^240^ PGE2 activates the EP4 receptor on sensory nerves and signals to the hypothalamus, downregulating sympathetic tone by activating the transcription factor cAMP response element-binding protein. Consequently, the adipogenic differentiation of BMSCs is inhibited, and osteoblastic differentiation is enhanced. Disruption of PGE2/EP4 signaling significantly impairs bone regeneration.^171,241^ Clinical administration of NSAIDs to reduce pain delays fracture healing and increases bone nonunion, providing further supporting evidence.^242^ Similarly, treatment with opiates, another effective class of analgesic drugs whose receptors are widely expressed on central and peripheral nerves,^243^ also leads to impaired bone healing.^244,245^ These clinical observations complicate pain management after the bone fracture, urging more intensive studies to support clinical strategies. Given the critical role of the inflammatory environment after bone trauma, this pathway holds great importance in the regenerative process. Although PGE2 is not released by neurons, neuro-skeletal regulation is initiated by PGE2. Moreover, direct effects of PGE2 on bone-lineage cells have also been reported.^246–248^ PGE2 signaling indicates that sensory and sympathetic nerves act as a circuit, and the collaboration of sensory and sympathetic nerves as parallel efferent regulators to maintain hematopoietic stem cells in BM niches has also been reported recently.^249^ The full picture of the relationship between sensory and sympathetic nerves in bone regeneration has yet to be revealed. Great progress has been made in identifying the roles of neuropeptides and neurotransmitters in bone regeneration, but the exact mechanisms whereby neuropeptides and neurotransmitters regulate specific bone regenerative processes are still under investigation (Fig. 5).

### SCs and endoneurial mesenchymal cells

SC secretion has been given the greatest attention in peripheral nerve regeneration.^8^ Paracrine effects also contribute to the function of SCs during bone regeneration. Transplantation of SCs into the denervated mandibular defects of mice effectively mitigates impaired defect regeneration, resulting in significantly increased bone formation.^25^ PDGF-AA, oncostatin M, and parathyroid hormone have been shown to promote bone formation following the implantation of SCs in denervated mice.^25^ The paracrine effect is also substantiated by the observation that conditioned medium from SCs promoted the proliferation and migration of BMSCs and improved fracture healing.^250^ Exosomes derived from SCs showed a similar promoting effect on BMSCs and bone regeneration.^251^

Furthermore, a developmental link between the nervous and skeletal systems through SCs has been established.^252^ SC precursors (SCPs) detach from nerve fibers and differentiate into chondrocytes and mature osteocytes.^253^ SCs detach from injured nerves and dedifferentiate, exhibiting a phenotype that mimics that of stem cells. Using a clonal color-coding technique to trace peripheral glia showed that SCPs and SCs were dormant NCCs that could be recruited from injured nerves.^253^ In addition, SC-derived dental MSCs can ultimately differentiate into odontoblasts, contributing to tooth regeneration after damage.^253^ Whether adult SCs directly contribute to bone formation in other parts of the skeletal system is a question that needs further investigation.

As previously described, cells reside in peripheral nerves that actively participate in regeneration (Fig. 3a). Recent single-cell RNA sequencing identified the cells in nerve fibers: SCs, fibroblasts, immune cells, and vasculature-associated cells.^254^ SCs and endoneurial fibroblasts are believed to be the main cells involved in PNS regeneration.^254^ A recent experiment identified a group of PDGFRα^+^ mesenchymal cells, including NCCs, in the endoneurium that displayed characteristics of mesenchymal precursor cells and could dramatically proliferate and differentiate into osteoblasts in vitro and in vivo after nerve injury, promoting regeneration of the digit tip.^55^

Neuropeptide interactions are noteworthy, and communication between CGRP and SP has also been reported. Bisphosphonates (BPs), which are typically used to treat certain bone diseases, can impair bone regeneration in the jaw by inhibiting osteoclastic bone resorption.^255^ BPs disturb the neuropeptide balance of CGRP and SP by decreasing CGRP levels and increasing SP levels in the callus. However, the administration of CGRP or SP alone had no effect on the BP-mediated decrease in a macrophage-like cell line (RAW 264.7 cells) in vitro, while concomitant application reduced toxicity.^256^ The ratio of CGRP to SP is tightly correlated with BP administration.^256^ SP or CGRP alone promotes BMP-2 signaling in MC3T3 preosteoblasts, but when combined, these factors inhibit osteogenic differentiation.^257^

**Fig. 6:**
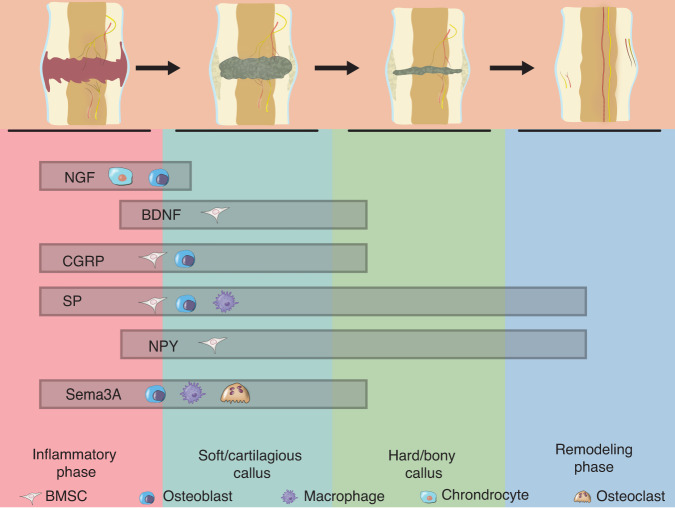
Increased production of neuropeptides in bone healing. Various neuropeptides (NGF, BDNF, CGRP, SP, NPY, and Sema3A) are differentially distributed during the four corresponding phases (inflammatory, soft/cartilaginous callus, hard/bony callus, and remodeling) of bone healing with blood vessel and nerve regeneration. Cells (BMSCs, osteoblasts, macrophages, osteoclasts, and chondrocytes) listed in the relevant boxes have been identified as targets of specific neuropeptides during bone regeneration

It should be noted that many neuropeptides and neurotransmitters released by the PNS after bone trauma can also be produced by nonneuronal cells, such as osteoblast-derived CGRP,^258^ osteocyte-derived SP,^108^ osteocyte-derived NPY,^194^ and callus-derived NGF.^259^ In particular, macrophage-derived NGF stimulates the ingrowth of skeletal sensory nerves as a key mediator of cranial bone regeneration.^132^ These factors could function as supplements to neuro-skeletal regulation or via a feedback loop, but the exact effect remains to be clarified. Therefore, conclusions on the function of the PNS in skeletal regeneration should be made with caution. The increased production of neuropeptides during bone healing is shown in Fig. 6, showing differential distributions in the inflammatory, soft/cartilaginous callus, hard/bony callus, and remodeling phases. These bioactive molecules produced by bone-lineage cells during regeneration will not only act on the PNS but also the vasculature and immune system and coordinate bone regeneration processes.

## Neurovascular interactions in bone regeneration

The vasculature develops prior to the nervous system during early embryonic development.^260^ However, studies in developing limb skin during later embryonic stages show that the alignment of nerve and blood vessels occurs via mesenchymal intrinsic cues or by blood vessels following the routes of peripheral nerves,^261–263^ which is consistent with observations in the fracture callus in bone repair. Neurovascular interactions have been indicated as early as the embryonic period, when nerves and vessels are guided by shared signals such as NGF, VEGF, and four classic axon guidance family proteins (semaphorins, ephrins, netrins, and slits) and corresponding receptors to their destinations.^88^ Similar pathfinding is mediated by common molecular cues and wiring patterns between blood vessels and sensory nerves. VEGF is released from sensory nerves or SCs and guides blood vessel formation locally during embryonic development and tissue repair. An association between neurovascular interactions and various pathologies, such as tumors^21,264^ and osteoarthritis (OA) (see below), has also been noted. Recently, precisely orchestrated neurovascular communication has been indicated in the context of bone regeneration, further verifying and extending the molecular portfolio involved in this interaction.

The anabolic effect of neuropeptides occurs through direct binding to bone-lineage cells and through their effects on endothelial cells. Close contact between sensory neurons and bone marrow-derived endothelial cells in cocultures has been observed.^265^ The administration of CGRP and SP, which are two important neuropeptides released by sensory nerves, upregulates VEGF, type 4 collagen, and matrix metalloproteinase 2.^265^ Interestingly, Mg^2+^ can induce CGRP to phosphorylate focal adhesion kinase, increasing VEGF expression and vessel and bone formation.^266,267^ CGRP also increases the number of endothelial progenitor cells differentiated from BMSCs in vitro via the PI3K/AKT signaling pathway and promotes blood vessel formation at defect sites in a DO model.^268^ The role of neurotrophins in neurovascular interactions during bone regeneration has also been reported. Inhibiting NGF/TrkA signaling blunts revascularization during bone regeneration, as shown by reduced numbers of CD31^+^ vessels within fracture sites.^123^ Systemic treatment with NT-3 immunoneutralization suppresses vascularization at the injury site, while recombinant NT-3 potentiates the expression of VEGF and CD31 in rat endothelial cells.^206^ The regulatory effect of osteoblast-derived SLIT3 on level of CD31^hi^EMCN^hi^ skeletal endothelial cells is mediated by SLIT3/ROBO1 pathway, which actively participate in osteogenesis, promotes bone formation,^269^ and the common pathway of SLIT3 may contribute to the alignment of the reconstructed nerve fibers and newly formed blood vessels during bone regeneration. In addition, deletion of *Slit3* in mice significantly impaired bone regeneration, and intravenous injection of SLIT3 in mice led to improved vascularization of the fracture callus and biomechanical properties.^269^ SC-conditioned medium can promote the proliferation, migration and tube formation of endothelial cells derived from BM-MSCs.^250^ Taken together, this evidence suggests that peripheral nerves are closely involved in angiogenesis during bone regeneration, but the questions of when and how this aligned pattern of nerves and blood vessels is recovered in complex but sequential bone regenerative processes remain unanswered.

## Neuroimmune interactions during bone repair/regeneration

The nervous system and immune system are traditionally thought to work independently, but the role of the nervous system in the host immune response to infection or injury has led to more new discoveries. The expression of Toll-like receptors, which were previously thought to be specific to the immune system, has been identified on sensory nerve fiber terminals.^265,266^ The sensitization of sensory nerves in the inflammatory phase during bone regeneration, as described previously, has suggested additional shared molecules, such as TNF, IL-1β, and IL-17A.^121^ The nervous system communicates with the immune system through neurotransmitter receptors, such as muscarinic and nicotinic acetylcholine receptors and α- and β-adrenergic receptors, which usually act on the nervous system but have also been identified on macrophages, dendritic cells, T and B lymphocytes, and even endothelial cells.^267^ The concept of the neuroimmune cell unit, which refers to a place in which immune and neuronal cells are present and closely communicate, provides further clarity.^270^

Emerging studies on neuroimmune regulation in bone regeneration have focused on the phenotypic switch from proinflammatory M1 to regenerative M2 macrophages. Macrophages are involved in all bone regenerative processes, but the underlying mechanisms that control the switch are still not clear, despite studies in this field.^271,272^ The axon reflex may contribute to the neuroimmune interaction in which sensory nerves initiate the transmission of neural signals at the fracture site, and then this neuronal activation reverses to local axonal terminals before being received by the CNS. This will increase the release of neuropeptides. Receptors of SP and CGRP have been detected on mouse BMMs.^158,273^ After M1 activation induced by lipopolysaccharide and IFN-γ, BMMs harvested from CGRP-deficient mice showed higher expression of the M1 macrophage marker CD86 and lower expression of the M2 macrophage marker CD206 than BMMs from wild-type mice, and the expression of M1-associated factors, such as TNF-α, iNOS, and IL-1, was also increased. Supplementation of CGRP in CGRP-deficient BMMs in vitro reversed these changes.^274^ CGRP lentiviral vector transfection into *CGRP*^−/−^ mice promoted the expression of M2-associated markers, such as Arg1 and CD206, in recruited macrophages at the injury site after tooth extraction.^274^ Moreover, inferior alveolar nerve transection prior to tooth extraction increased the number of recruited neutrophils and reduced the cellular elongation of macrophages, which is associated with the M1 phenotype,^275^ creating a proinflammatory environment.^276^ Implantation of CGRP-loaded microbeads into the socket after tooth exaction increased the expression of IL-10 and suppressed TNFα expression in macrophages. Local macrophage blocking by anti-F4/80 antibodies virtually eliminated the previous CGRP-induced effect.^276^ Recent work shows that CGRP can regulate the secretion of many osteogenic factors in M2 macrophages and promote the osteogenic differentiation of MSCs by activating p-Yap1 in M2 macrophages.^277^

The CNS mediates reflexes in which responses generated in the CNS travel down to the autonomic nerve fiber terminals. In a clinically relevant mouse model of PTSD, mice have increased numbers of Ly6G^+^ neutrophils in the fracture hematoma and later callus, and this effect could be ameliorated by treatment with propranolol to improve impaired fracture healing.^176^ Postoperative administration of donepezil significantly lowers the infiltration of lymphocytes and macrophages but hinders new bone formation.^186^ These results indicate that ANS regulates recruitment in the inflammatory phase of bone regeneration. Moderate inflammation in bone regeneration has been shown to be necessary for bone regeneration. The observation of neuroimmune interactions in bone regeneration provides initial evidence for a new immune regulator in the skeleton. However, these results are far from clarifying the cellular or molecular mechanisms of neuroimmune cell interactions and the specific role of this interplay during bone regeneration.

The aforementioned observations are partly consistent with the neuroimmune regulation seen in other organs. In collaboration with macrophages, CGRP can regulate the activation of PKA, reduce TNF-α production, and induce the expression of IL-10 in skin wounds.^109^ TRPV1^+^ nerves suppress immune activity by releasing CGRP.^278^ Other neuropeptides associated with neuroimmune regulation, including SP and VIP, have also been identified in other physiological or pathological processes and are well documented.^109,120^ The necessity of the “nerve, immune, bone” triad is demonstrated by the association between injured nerves, macrophage-derived NGF, and bone regeneration.^132^ Further examination of neuroimmune regulation in the unique bone niche should continue.

## Participation of peripheral nerves in other bone disorders

A large body of preclinical data suggests multiple functions of peripheral nerves in bone pathophysiological conditions, including regenerative processes after injury. Peripheral nerves have been suggested to participate in osteoporosis, OA, bone-related tumors (especially bone metastasis),^279^ and bone changes related to psychosomatic illness.^280,281^ The interactions between peripheral nerves and cells in the bone microenvironment in different bone diseases may provide a holistic perspective of the functions of peripheral nerves in bone.

Osteoporosis is usually characterized as an aging-related endocrine disease. In fact, aging can function as a separate process with distinct mechanisms that shape the bone environment, as connecting aged mice to a youthful circulation via heterochronic parabiosis did not improve aging-induced bone loss.^282^ In addition to senescent cells,^283^ changes in nerves in bone with aging have also been noted. Leptin, a hormone that is found exclusively in adipose tissue and regulates food intake, has been discovered in hypothalamic centers and is associated with bone loss in obese mice, supporting the close link between the nervous system and bone homeostasis.^284^ Further research showed that low sympathetic tone resulting from leptin deficiency increased bone mass by regulating the proliferation of osteoblasts and the expression of the osteoclast differentiation factor RANKL in osteoblasts.^144,173^ Poor peripheral nerve function is also related to lower bone mineral density in patients.^285^ In osteoporosis and aging-related osteopathic disorders, innervation in bone is impaired. The peroneal nerve, which innervates the lower leg, has increased sympathetic tone in osteoporosis patients and is inversely correlated with bone quality.^286^ Reduced nerve fibers in the tibia are reported in mice with ovariectomy-induced bone loss and reduced expression of neuronal factors and neurotransmitters.^287^

OA is a chronic degenerative joint disease characterized by excruciating pain. Increased nerve ingrowth along with newly formed blood vessels in the synovium, osteophytes, and menisci are thought to be associated with OA development and progression.^288^ OA pain is related to increased sensory nerve fibers, and increased levels of factors such as neuropeptides, VEGF, and NGF may also induce additional pain sensation.^288^ Osteoclasts can release netrin-1 to induce the growth of sensory nerves and lead to pain in OA.^239^ Despite many new findings on anti-NGF therapy, the roles of the PNS in rapidly progressive OA and OA with severe joint degeneration require further investigation, as do the serious adverse effects of treatment.^289^

## Conclusion

Bone regeneration is a nerve-dependent process. Continuous discoveries regarding the roles of the PNS in bone repair and regeneration shed light on musculoskeletal biology. Neurotransmitters, neuropeptides, and nerve cell redifferentiation are the main features of neuro-skeletal regulation and have complex molecular mechanisms. New technologies allow in-depth investigations of the interplay of nerves and various cells in the bone at different stages of repair and regeneration.

With further understanding of the multiple functions of nerves in bone homeostasis and regeneration, new therapies to promote nerve-bone interactions will significantly improve bone repair management outcomes and reduce patients’ pain and suffering.

## Supplementary information

Editing certification
