# Supplementary figures and images for: Hallmarks of peripheral nerve function in bone regeneration
Source: Bone Res. 2023 Jan 5;11:6. doi: 10.1038/s41413-022-00240-x (PMC9813170; doi:10.1038/s41413-022-00240-x)

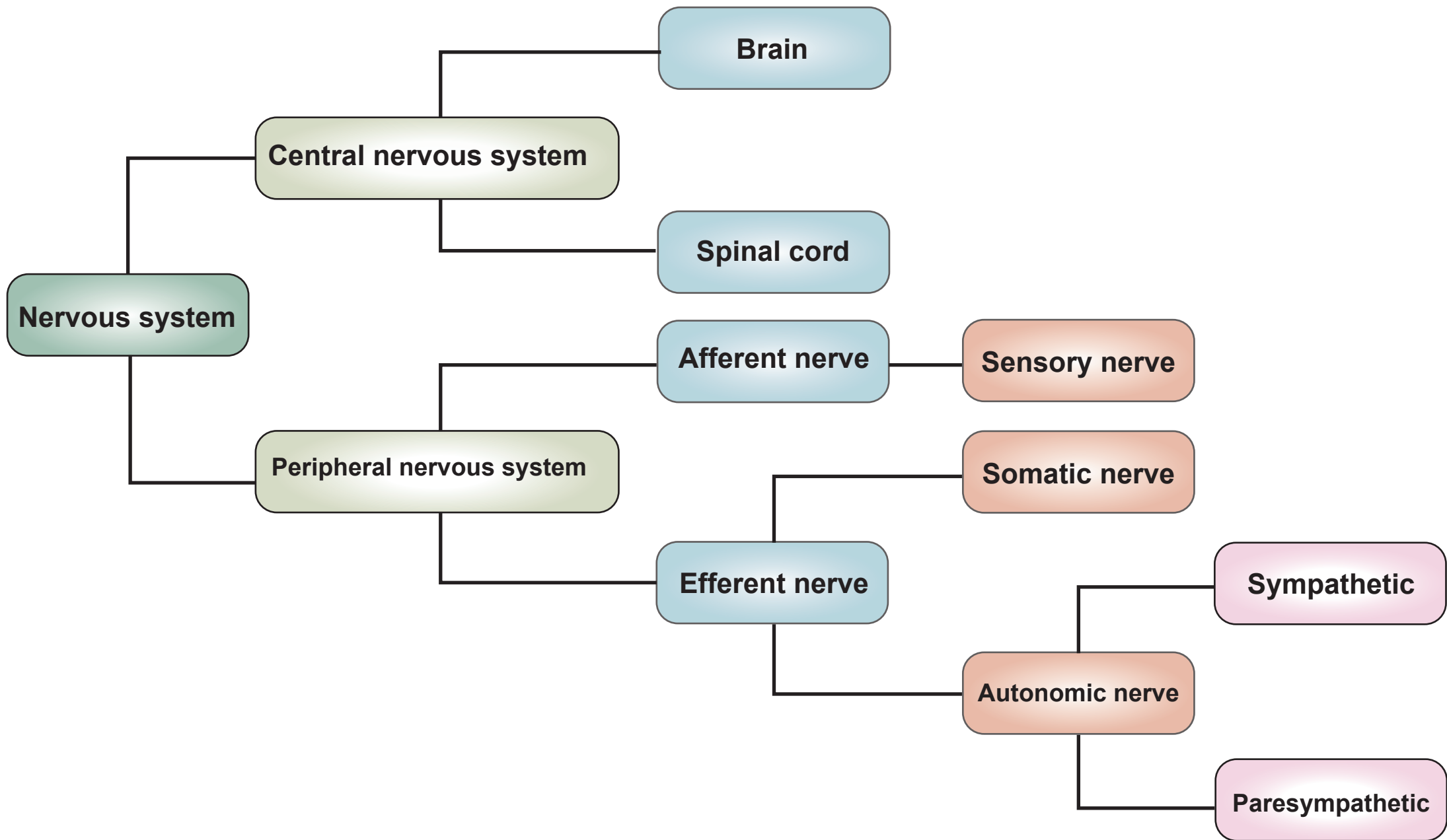

Supplement: Supplementary file 1 — Editing certification [file 41413_2022_240_MOESM1_ESM.pdf]
